# Emotional State of Being Moved Elicited by Films: A Comparison With Several Positive Emotions

**DOI:** 10.3389/fpsyg.2019.01935

**Published:** 2019-08-20

**Authors:** Kenta Kimura, Satoshi Haramizu, Kazue Sanada, Akiko Oshida

**Affiliations:** ^1^Automotive Human Factors Research Center, National Institute of Advanced Industrial Science and Technology (AIST), Tsukuba, Japan; ^2^Kao Corporation, Kansei Science Research, Tokyo, Japan

**Keywords:** positive emotion, being moved, film, autonomic nervous system, facial expressions

## Abstract

This study investigated the facial and physiological activities that are associated with the emotional state of being moved. We elicited the emotional states of being moved, amusement, attachment, and calmness by presenting participants with film clips; we assessed their electromyographic, electrodermal, and cardiac responses to the films. Further, we used a high- and low-arousal moving film to examine the effect of different levels of arousal on facial and physiological responses to moving films. We compared facial and physiological responses to positive-emotion and emotionally neutral films. Analysis of subjective emotion scale ratings revealed that the films had successfully elicited the target positive emotions and that the high- and low-arousal moving films had elicited the feeling of being moved in accordance with the anticipated level of intensity. In comparison to the other types of positive-emotion films, the two moving films resulted in an increase in corrugator electromyography activity and skin conductance responses, which in turn were modulated by the arousal level of the moving films. However, cardiac measures such as heart rate did not differ across the different film conditions. These results suggest that film clips can elicit different intensities of the emotional state of being moved and that facial muscle and electrodermal activities but not cardiac activity characterize the film-induced emotional state of being moved.

## Introduction

Emotions are multicomponential responses that include coordinated changes in subjective feelings, behaviors, and physiological activities (e.g., Frijda, 1986, 1993; Scherer and Moors, 2019). Most previous studies on emotions have mainly focused on emotional responses such as facial muscle movements and autonomic nervous activities that are associated with negative emotions (e.g., fear, anger). These studies have found that discrete negative emotional responses are highly differentiated (e.g., Cacioppo et al., 2000; Friedman, 2010). In contrast, the question of whether discrete positive emotional responses are differentiable has received substantially lesser attention. However, recent studies have begun to address this issue (for a review, see Shiota et al., 2017).

### Association Between Positive Emotions and Emotional Responses

Previous studies have examined the facial and physiological activities that are associated with several positive emotions (for a review, see Kreibig, 2010). Traditionally, positive emotions have been linked to one expressive display, namely, the Duchenne smile (e.g., Ekman, 1992), which is characterized by increased activation of the zygomaticus major and orbicularis oculi muscles. However, recent studies have shown that positive emotions are not necessarily associated with smiles. For instance, Campos et al. (2013) examined the expressive display patterns of eight emotional states: amusement, awe, contentment, gratitude, interest, joy, love, and pride. They found that amusement, joy, contentment, love, and pride resulted in smiles that varied in intensity, whereas awe and interest did not result in smiles. The findings of this and other studies suggest that positive emotions are not necessarily associated with smiles but may be associated with divergent patterns of facial muscle activity (e.g., Shiota et al., 2003). Similarly, positive emotions can also be associated with divergent patterns of physiological activity (for a review, see Kreibig, 2010). For instance, it has been reported that happiness is associated with an increased (e.g., Neumann and Waldstein, 2001), an unchanged (e.g., Levenson, 1992), or a decreased (e.g., Krumhansl, 1997) heart rate (HR). These findings suggest that positive emotional states are not necessarily associated with one specific emotional response pattern but may be associated with divergent patterns of facial and physiological activities.

Previous studies have shown that divergent patterns of facial and physiological activities are attributable to differences in the method of emotion induction (e.g., Cacioppo et al., 2000; Stemmler et al., 2001). Specifically, previous studies have reported that the method of emotion induction itself has an effect on facial and physiological activities (e.g., McGinley and Friedman, 2017). In other words, the different types of stimuli that are used to induce emotions (e.g., images, music, film clips) require varied attentional and cognitive processes, which in turn may have unique effects on facial and physiological activities. Previous studies have demonstrated that attentional and cognitive processes have an impact on facial and physiological activities (e.g., van Boxtel and Jessurun, 1993; Waterink and van Boxtel, 1994; Overbeek et al., 2014). For instance, facial muscle activities are influenced not only by emotional components but also by a large variety of activities that are unrelated to emotional components such as mental effort, task involvement, and anticipation of sensory stimuli (for a review, see van Boxtel, 2010). Further, it is widely known that the orienting effect of attention to external stimuli (e.g., images, film clips) can cause changes in a variety of physiological activities (e.g., Sokolov, 1990; Stekelenburg and van Boxtel, 2002; Bradley, 2009). From this standpoint, any effect of positive emotions must be considered alongside the activities that result from emotion induction (e.g., Christie and Friedman, 2004; McGinley and Friedman, 2017).

Several positive emotional states have been investigated to date. These include affection, amusement, contentment, and happiness (for a comprehensive review, see Kreibig, 2010, 2014). However, only a few studies have examined the emotional state of being moved. Menninghaus et al. (2015) have recently proposed that the emotional state of being moved is associated with both a positive and negative valence and that such states are elicited by critical life and relationship events including deaths, births, friendships, and parent–child interactions. Tokaji (2003) found that joy and sadness are the two preeminent emotions that are involved in the experience of being moved. This suggests that the emotional state of being moved can be categorized as a mixed emotional state (e.g., Menninghaus et al., 2015; for a review, see Zickfeld et al., 2019).

To date, only a few studies have examined the emotional responses that are associated with the experience of being moved. Some studies have examined the relationship between the experience of being moved and bodily sensations using self-report measures (for a review, see Zickfeld et al., 2019). This line of research has found that the experience of being moved is associated with specific bodily sensations such as tears (e.g., Tan and Frijda, 1999), a warmth in the center of the chest (e.g., Schubert et al., 2016), or the experience of chills (e.g., Koelsch, 2010). However, these studies have used self-report measures but not facial and physiological measures. Facial and physiological activities that are associated with the emotional state of being moved have been partially examined within the context of music-induced chills (for a review, see Koelsch, 2010). Previous studies have reported that the chills that are induced by auditory stimuli (i.e., music and the recitation of poems) are associated with increases in corrugator muscle activity (e.g., Wassiliwizky et al., 2017), HR, electrodermal activity, and respiratory rate, thereby implicating the involvement of sympathetic nervous activation (Craig, 2005; Guhn et al., 2007; Grewe et al., 2009; Salimpoor et al., 2009, 2011; Mori and Iwanaga, 2014, 2017). Since chills are closely related to the emotional state of being moved (for a review, see Koelsch, 2010), these findings may be interpreted as implying that the emotional state of being moved is associated with an increase in corrugator muscle and sympathetic nervous activities.

### The Present Study

Although facial and physiological activities that are associated with the emotional state of being moved have been partially investigated in relation to the experience of chills, the respective studies have certain limitations. First, previous studies have examined only facial and physiological activities that are associated with the occurrence of chills. Although chills are closely related to the emotional state of being moved (for a review, see Koelsch, 2010), they are not sufficient indicators of the emotional state of being moved (for a review, see Zickfeld et al., 2019). Therefore, it is necessary to directly examine the association between the experience of being moved and facial and physiological activities. Second, previous studies on the experience of chills have been mainly conducted within the domain of music, and most studies have used music to induce chills. However, as discussed earlier, the varied characteristics of different emotion induction methods have unique effects on facial and physiological activities (e.g., Christie and Friedman, 2004), which in turn can result in divergent patterns of facial and physiological activities that are associated with different emotional states (e.g., Cacioppo et al., 2000; Stemmler et al., 2001). This suggests that facial and physiological activities that are associated with the emotional state of being moved may differ in accordance with the method of emotion induction. Third, none of the past studies have compared the emotional state of being moved and other types of positive emotions. Therefore, it remains unclear whether the emotional state of being moved is different from other types of positive emotions.

The present study was designed to examine the aforementioned issues by inducing emotions using film clips. Film clips are widely used to elicit several emotions (for a review, see Rottenberg et al., 2007) including mixed emotions (e.g., Hemenover and Schimmack, 2007; Kreibig et al., 2013), and the resultant emotions have been validated against self-reports. Film clips can also be used to elicit the facial and physiological responses that are associated with positive, negative, and mixed emotions (for a review, see Kreibig, 2010; Kreibig and Gross, 2017). In the present study, the emotional state of being moved was elicited by presenting participants with film clips while electromyographic (EMG), electrodermal, and cardiac activities were assessed. We used two moving films (i.e., movies with a moving storyline), namely, a high- and low-arousal film, to examine whether the level of arousal influences facial and physiological responses to moving films. Furthermore, we compared facial and physiological responses to moving films using films that also elicited other types of positive emotions such as amusement, attachment, and calmness to examine whether the elicited emotional state of being moved is distinct from the other types of positive emotions. These specific types of positive emotions were chosen because their facial and physiological responses have been relatively well characterized in the existing literature (for a review, see Kreibig, 2010; Shiota et al., 2011; Nittono and Ihara, 2017). This allowed us to compare the emotional state of being moved and other types of positive emotions. In accordance with Shiota et al. (2011) suggestion, we compared facial and physiological responses to positive-emotion films (i.e., those that were specific to the aforementioned positive emotions) and an emotionally neutral film. If the emotional state of being moved varies across different emotion induction methods, then facial and physiological responses to the two moving films must differ from those that have been reported in previous studies on music-induced chills (i.e., increases in corrugator muscle activity, HR, and electrodermal activity) (e.g., McGinley and Friedman, 2017). Furthermore, if the emotional state of being moved that is elicited by film clips is distinct from other types of positive emotions, then facial and physiological responses to the two moving and other positive-emotion films must be different (e.g., Shiota et al., 2011).

## Materials and Methods

### Participants

Twenty-eight adults (women: *n* = 16, men: *n* = 12; age range = 20–36 years, *M*_*age*_ = 22.36 years) participated in this study. They were recruited through social media and by email based on contact information that had been stored in a research database. The final sample size was ascertained based on the sizes of samples that were used in previous studies that have investigated facial and psychophysiological responses to emotional films (for a review, see Kreibig, 2010). All the participants had normal or corrected-to-normal vision and had no history of neurological or mental disorders. Written informed consent was obtained from all the participants as per the protocols that have been formulated by the Safety and Ethics Committee of the National Institute of Advanced Industrial Science and Technology (AIST).

### Stimuli and Apparatus

We used six types of film stimuli that depicted the following emotions: high-arousal moving (HA-MOV), low-arousal moving (LA-MOV), amusement (AMUSE), attachment (AT), calmness (CAL), and neutral (NE) emotions. The film stimuli were selected based on the criteria that have been recommended by Gross and Levenson (1995): length, intelligibility, intensity, and discreteness. In the pilot study, 12 participants were asked to watch the films and rate their subjective emotional feelings on discrete (amusement, feeling moved, attachment, and calmness) and dimensional (valence and arousal) emotion scales. The results showed that the target emotions were successfully elicited in each emotion condition. The film stimuli varied in duration from 94 to 183 s (*M* = 133 s). The durations of the film stimuli were varied to ensure that the content is clear, sensible, and facilitative of a progression toward the climax. All the film stimuli except the neutral film stimulus had audio content.

The HA-MOV film was a drama about a doctor who saves the life of a sick man who had provided him with food and shelter when he was a young boy. The LA-MOV film was a drama that depicted how a man apologizes to his grandmother for hurting her in the past and realizes the preciousness of familial bonds. The AMUSE film entailed a comedy routine about a young man who hits the wrong target while fencing. The AT film showed a baby who was laughing and sleeping. The CAL film showed natural scenes of flowers in a park. The NE film was a non-commercial screensaver that has been recommended by Rottenberg et al. (2007). The contents of the films were similar to those that have been described in previous research reports (e.g., Gross and Levenson, 1995; Rottenberg et al., 2007; Ge et al., 2018), thereby suggesting that the films are representative of each target emotion. None of the participants were familiar with any of the film stimuli.

The presentation of visual and auditory stimuli was controlled using a Presentation software (Neurobehavioral Systems) that was installed on a laptop (Lenovo, ThinkPad W540). All visual stimuli were presented on a 22-inch LCD monitor (Dell, E2210) at a viewing distance of 60 cm under low ambient illumination. The audio content of the film stimuli was transmitted through headphones (Audio-Technica, ATH-AD900X) using an audio interface (Steinberg, UR44). The intensity of the transmitted sound was set to a comfortable listening level prior to the commencement of the experimental session.

### Procedure

The participants were individually tested in a laboratory. After they arrived at the laboratory, they were briefly informed about the study, following which they signed an informed consent form. Next, the participants were seated in front of a computer screen, and the sensors for the physiological measures were attached. To conceal the fact that their facial muscle activities were being recorded, the participants were led to believe that their skin temperatures were being measured by the sensors that were placed on their facial muscles. After the sensors were attached, the participants were asked to remain seated on the chair and relax for 5 min while keeping their eyes open to habituate them to the laboratory and sensors. After the resting period had ended, the six film stimuli were presented. The order of film presentation was pseudorandomized across participants. The presentation of each film was preceded by a 2-min baseline period and followed by a 2-min recovery period. The durations of the baseline and recovery periods was determined in accordance with those that have been used in previous studies that have used emotional films (e.g., Gross and Levenson, 1997; Kreibig et al., 2007) to limit carryover from one film to the next (Rottenberg et al., 2007). During the baseline and recovery periods, the words “Relax on a chair” were displayed at the center of the monitor, and the participants were asked to relax with their eyes open. The experimenter entered the room at the end of the recovery period of each film presentation and administered a questionnaire that assessed emotional feelings.

### Measures

#### Subjective Ratings

The participants rated the extent to which they felt moved, amused, attached, and calm when they watched the film clips on a discrete emotion scale that entailed a 9-point scale, which ranged from 0 (*not at all*) to 8 (*very strong*). The target words were “being moved,” “amused,” “attachment,” and “calm” for the HA-MOV/LA-MOV, AMUSE, AT, and CAL films, respectively. The participants also rated the valence and arousal of the emotions that were elicited by the film clips on a dimensional emotion scale that entailed a 9-point scale. The response anchors ranged from 0 (*unpleasant*) to 8 (*pleasant*) for valence and 0 (sleepy) to 8 (*highly aroused*) for arousal.

#### Facial and Physiological Measures

All physiological signals were recorded using a digital amplifier (Brain Products, BrainAmp ExG) and a software package (Brain Products, Brain Vision Recorder). The facial EMG activities of the corrugator supercilii and zygomaticus major muscles were measured using Ag/AgCl electrodes with a 4-mm-diameter Ag/AgCl detection surface that was placed over the corrugator supercilii and zygomaticus major muscles of the left side of the face. The interelectrode distances (center-to-center) were approximately 12 mm. The impedance of all the electrodes was reduced to less than 10 kΩ. The raw EMG signals were bandpass filtered at 10–1000 Hz and digitized at a sampling rate of 1000 Hz. The signals were subjected to 50-Hz notch filtering, 30–400-Hz digital bandpass filtering, rectification, and were smoothed with a time constant of 200 ms (cf. van Boxtel, 2010).

Electrocardiograms (ECGs) were recorded using Ag/AgCl electrodes in a standard lead II configuration. The ECG signals were bandpass filtered at 0.1–1000 Hz and digitized at a sampling rate of 1000 Hz. R-peaks were detected using a peak detection function (Billauer, Version 3.4.305) in MATLAB (MathWorks, Inc., MATLAB2010b). R-peaks were checked for artifacts and ectopic beats, and they were corrected when necessary. Subsequently, RR intervals (ms) were converted into HR (bpm).

Skin conductance responses (SCRs) were measured using two Ag/AgCl disposable electrodes that were filled with isotonic electrolyte paste and placed on the palmar side of the distal phalanges of the index and middle fingers of the left hand. The electrodes were connected to a skin conductance level/response unit (Vega Systems, DA-3b) that imposed a constant voltage of 0.5 V across them (Fowles et al., 1981). A high-pass filter of 0.04 Hz was applied to the recording. As recommended by Shiota et al. (2011), we counted the number of non-specific SCRs that were recorded during film viewing. Valid SCRs were defined as increases of at least 0.05 μS that had occurred when the participants were viewing the films. The total number of valid SCRs that were recorded during each film viewing epoch was counted. SCRs that had occurred for less than 5 s after the commencement of the film clip were not included in the total count in order to exclude the SCRs that had been elicited by the commencement of film presentation. The SCR data of four participants (2 men and 2 women) were excluded because they contained excessive recording artifacts.

### Data Analysis

#### Subjective Ratings

In order to examine whether the film stimuli had elicited the target emotions, we conducted planned comparisons of the target and each non-target emotion on discrete emotion scale ratings using two-tailed paired *t*-tests (Sato et al., 2007). One-way repeated-measures analyses of variance (ANOVA) were conducted to examine differences in the valence and arousal ratings of the dimensional emotion scale across the six film conditions: HA-MOV, LA-MOV, AMUSE, AT, CAL, and NE films. The Greenhouse-Geisser epsilon (ε) correction was applied when the data violated the assumption of sphericity. Effect sizes were estimated by computing partial eta squared (${\text{η}}_{\text{p}}^{2}$) values. Planned comparisons were executed using two-tailed paired *t*-tests. The level of significance was set as 0.05.

#### Facial and Physiological Measures

Physiological data were processed using a customized biosignal analysis software that was written in MATLAB. Period averages were computed for each baseline and film presentation period (e.g., Kreibig et al., 2013). With regard to corrugator and zygomaticus EMG activities, change scores were quantified as a percentage of the mean EMG level during the baseline period immediately preceding each film stimulus (e.g., van Boxtel, 2010). Change scores for HR were calculated by subtracting the average score for the immediately preceding baseline period from the average score for the subsequent film presentation period. For SCRs, we used the total number of valid SCRs that were recorded during each film presentation period (Shiota et al., 2011). The significance of the difference between these scores and their respective baseline scores was tested using two-tailed paired *t*-tests for each film condition. Further, one-way repeated-measures ANOVA was conducted to examine differences in the aforementioned scores across the six film conditions: HA-MOV, LA-MOV, AMUSE, AT, CAL, and NE. The Greenhouse-Geisser epsilon (ε) correction was applied when the data violated the assumption of sphericity. Effect sizes were estimated by computing partial eta squared (${\text{η}}_{\text{p}}^{2}$) values. We conducted specific planned comparisons using two-tailed paired *t*-tests to compare (a) the NE condition against each positive-emotion film condition and (b) the HA-MOV and LA-MOV conditions. The level of significance was set as 0.05.

## Results

### Subjective Ratings

Table 1 shows the means for the discrete emotion scale ratings in each film condition. Planned comparisons between the target and each non-target emotion in each film condition were conducted. In the HA-MOV condition, the ratings for feelings of being moved were significantly higher than those for all the other emotion words [*t*s(27) = 11.76, 7.48, and 6.61, for amusement, attachment, and calmness, respectively; *p*s < 0.01]. Moreover, in the LA-MOV condition, the ratings for feelings of being moved were significantly higher than those for all the other emotion words [*t*s(27) = 7.81, 3.40, and 4.70 for amusement, attachment, and calmness, respectively; *p*s < 0.01]. Furthermore, in the AMUSE condition, the ratings for amusement were significantly higher than those for all the other emotion words [*t*s(27) = 12.48, 8.11, and 8.32 for feelings of being moved, attachment, and calmness, respectively; *p*s < 0.01]. Additionally, in the AT condition, the ratings for attachment were significantly higher than those for all the other emotion words [*t*s(27) = 10.20 and 6.34 for feelings of being moved and attachment, respectively; *p*s < 0.01], except calmness (*p* > 0.1). Finally, in the CAL condition, the ratings for calmness were significantly higher than those for all the other emotion words [*t*s(27) = 8.84, 9.36, and 7.68 for feelings of being moved, amusement, and attachment, respectively; *p*s < 0.01].

**TABLE 1:** Descriptive statistics for discrete emotion scale ratings in each film condition.

	***M* (*SD*)**			
**Film condition**	**Being moved**	**Amusement**	**Attachment**	**Calmness**
High arousal moving	7.04 (0.88)	2.71 (1.58)	3.75 (2.27)	4.18 (2.16)
Low arousal moving	6.61 (1.59)	3.32 (1.76)	5.29 (2.11)	5.51 (1.83)
Amusement	0.86 (1.33)	5.79 (2.15)	2.64 (2.16)	1.64 (1.57)
Attachment	2.68 (1.94)	4.07 (2.02)	6.00 (1.85)	5.86 (1.56)
Calmness	2.50 (2.03)	2.64 (1.99)	3.04 (2.10)	6.39 (1.59)
Neutral	1.54 (1.93)	2.07 (1.78)	0.96 (1.20)	2.04 (1.95)

**FIGURE 1:**
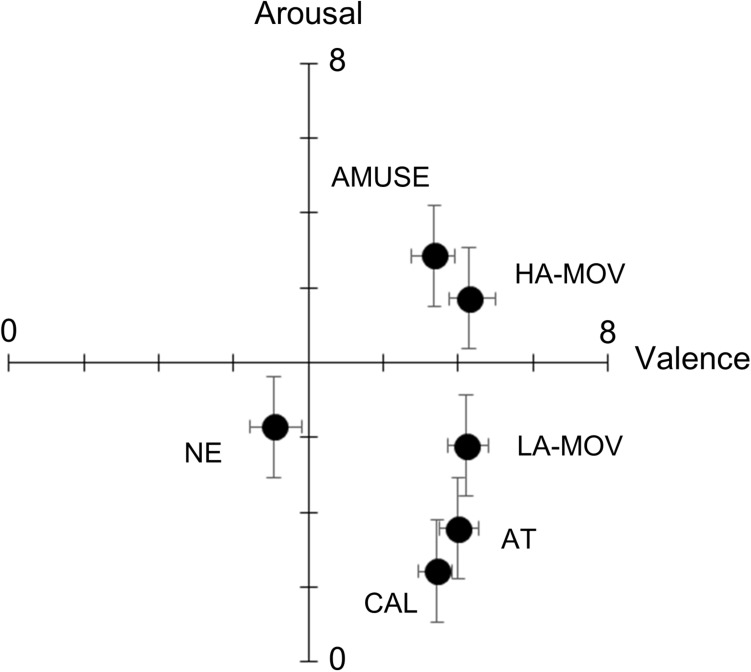
Mean (±SE) ratings for the dimensional emotion scales. HA-MOV, arousal moving; LA-MOV, low arousal moving; AMUSE, amusement; AT, attachment; CAL, calmness; NE, neutral.

Figure 1 shows the valence and arousal ratings in each film condition. One-way ANOVA (levels: 6 film conditions) was conducted with valence ratings as the dependent variable. There was a significant main effect for film condition [*F*(5,135) = 14.96, *p* < 0.01, ${\text{η}}_{\text{p}}^{2}$ = 0.36]. *Post hoc* comparisons revealed that valence ratings were significantly lower in the NE condition than in any other condition (*p*s < 0.01). No other comparison yielded statistically significant results (*p*s > 0.1). Next, one-way ANOVA (levels: 6 film conditions) was conducted with arousal ratings as the dependent variable. There was a significant main effect for film condition [*F*(5,135) = 31.97, *p* < 0.01, ${\text{η}}_{\text{p}}^{2}$ = 0.54]. Planned comparisons revealed that, when compared to the NE condition, arousal ratings were higher in the HA-MOV [*t*(27) = 3.24, *p* < 0.01] and AMUSE [*t*(27) = 2.29, *p* < 0.01] conditions and lower in the AT [*t*(27) = 3.06, *p* < 0.05] and CAL [*t*(27) = 5.42, *p* < 0.01] conditions. Arousal ratings were significantly higher in the LA-MOV condition than in the AT [*t*(27) = 3.16, *p* < 0.01] and CAL [*t*(27) = 6.16, *p* < 0.01] conditions. Conversely, arousal ratings were significantly lower in the LA-MOV condition than in the HA-MOV [*t*(27) = 4.58, *p* < 0.01] and AMUSE [*t*(27) = 5.79, *p* < 0.01] conditions. Arousal ratings did not differ significantly between the LA-MOV and NE conditions (*p*s > 0.1).

### Facial and Physiological Measures

**FIGURE 2:**
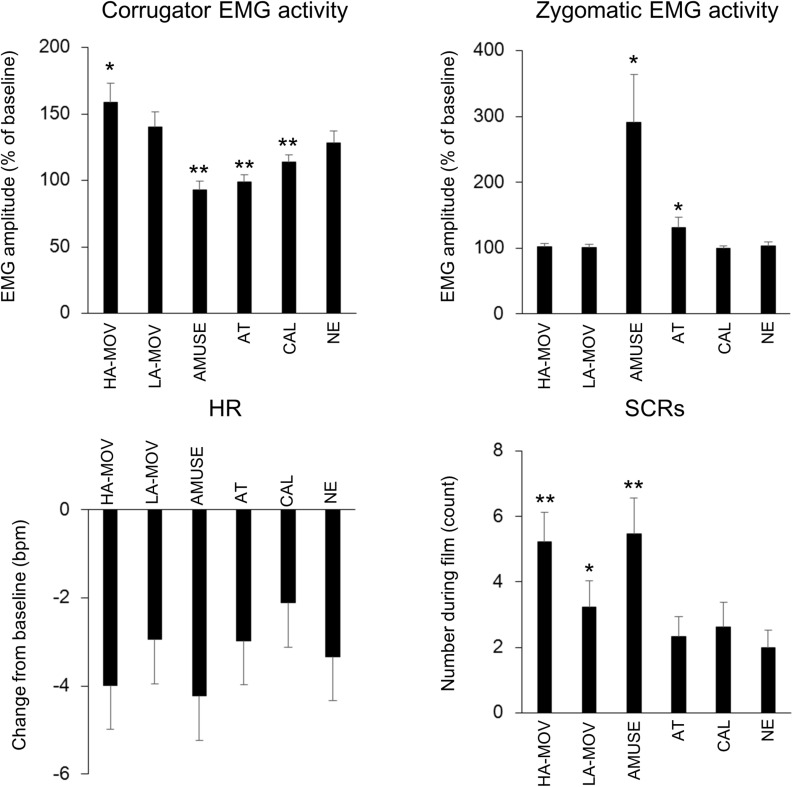
Means (±SE) for facial and physiological measures in each film condition. Asterisks show the significant differences from the NE condition: ^∗^*p* < 0.05, ^∗∗^*p* < 0.01.

Figure 2 shows the means for facial EMG activities, HR, and SCRs for each film condition. Corrugator EMG activity had significantly increased from baseline levels in the HA-MOV [*t*(27) = 4.18, *p* < 0.01], LA-MOV [*t*(27) = 3.63, *p* < 0.01], CAL [*t*(27) = 2.39, *p* < 0.05], and NE conditions [*t*(27) = 3.22, *p* < 0.01] but not the AMUSE and AT conditions (*p*s > 0.1). One-way ANOVA (levels: 6 film conditions) was conducted using the change scores for corrugator EMG activity. There was a significant main effect for film condition [*F*(5,135) = 8.57, *p* < 0.01, ε = 0.60, ${\text{η}}_{\text{p}}^{2}$ = 0.24]. Planned comparisons revealed that the change scores for corrugator EMG activity were higher in the HA-MOV condition [*t*(27) = 2.04, *p* < 0.05] and lower in the AMUSE [*t*(27) = 3.31, *p* < 0.01], AT [*t*(27) = 3.02, *p* < 0.01], and CAL [*t*(27) = 2.21, *p* < 0.05] conditions, when compared to the NE condition. The change scores for corrugator EMG activity in the LA-MOV condition did not differ significantly from those in the HA-MOV and NE conditions (*p*s > 0.1).

Zygomaticus EMG activity had significantly increased from baseline levels in the AMUSE condition [*t*(27) = 2.63, *p* < 0.01] but not the HA-MOV, LA-MOV, AT, CAL, and NE conditions (*p*s > 0.1). One-way ANOVA (levels: 6 film conditions) was conducted using the change scores for zygomaticus EMG activity as the dependent variable. There was a significant main effect for film condition [*F*(5,135) = 6.56, *p* < 0.05, ε = 0.22, ${\text{η}}_{\text{p}}^{2}$ = 0.20]. Planned comparisons revealed that the change scores for zygomaticus EMG activity were higher in the AMUSE [*t*(27) = 2.73, *p* < 0.05] and AT [*t*(27) = 2.09, *p* < 0.05] conditions than in the NE condition. The change scores for zygomaticus EMG activity in the HA-MOV and LA-MOV conditions did not differ significantly (*p* > 0.1).

HR had significantly decreased from baseline levels in the HA-MOV [*t*(27) = 5.44, *p* < 0.01], LA-MOV [*t*(27) = 4.43, *p* < 0.01], AMUSE [*t*(27) = 5.32, *p* < 0.01], AT [*t*(27) = 4.86, *p* < 0.01], CAL [*t*(27) = 4.16, *p* < 0.01], and NE [*t*(27) = 5.34, *p* < 0.01] conditions. One-way ANOVA (levels: 6 film conditions) was conducted with change scores for HR as the dependent variable. There was a marginally significant main effect for film condition [*F*(5,135) = 2.09, *p* = 0.09, ε = 0.71, ${\text{η}}_{\text{p}}^{2}$ = 0.07]. Planned comparisons revealed that the change scores for HR were lower in the CAL condition than in the NE condition [*t*(27) = 1.85, *p* = 0.08]. The change scores for HR in the HA-MOV and LA-MOV conditions did not differ significantly (*p*s > 0.1).

The number of SCRs had significantly increased from baseline levels in the HA-MOV [*t*(27) = 5.53, *p* < 0.01], LA-MOV [*t*(27) = 3.96, *p* < 0.01], AMUSE [*t*(27) = 5.04, *p* < 0.01], AT [*t*(27) = 3.63, *p* < 0.01], CAL [*t*(27) = 3.33, *p* < 0.01], and NE [*t*(27) = 3.56, *p* < 0.01] conditions. One-way ANOVA (levels: 6 film conditions) was conducted with the number of SCRs as the dependent variable. There was a significant main effect for film condition [*F*(5,115) = 6.31, *p* < 0.01, ε = 0.70, ${\text{η}}_{\text{p}}^{2}$ = 0.22]. Planned comparisons revealed that, when compared to the NE condition, the number of SCRs was higher in the HA-MOV [*t*(23) = 3.48, *p* < 0.01], LA-MOV [*t*(23) = 2.68, *p* < 0.05], and AMUSE [*t*(23) = 3.86, *p* < 0.01] conditions. Moreover, the number of SCRs was higher in the HA-MOV condition than in the LA-MOV condition [*t*(23) = 2.14, *p* < 0.05].

## Discussion

This study investigated the facial and physiological activities that are associated with the emotional state of being moved, which was elicited using film clips. Comparisons of the subjective ratings for target and non-target emotions showed that the ratings for target emotion words were significantly higher than those for non-target emotion words. This suggests that the anticipated target emotions were successfully elicited in each film condition. The results of the analyses that were conducted using dimensional emotion scale ratings showed that the valence ratings were higher in the HA-MOV, LA-MOV, AMUSE, AT, and CAL conditions than in the NE condition. However, the valence ratings did not differ between the HA-MOV, LA-MOV, AMUSE, AT, and CAL conditions. This indicates that all the emotion films were perceived to be equally positive. Arousal ratings differed between the different film conditions: high levels of arousal characterized the HA-MOV and AMUSE conditions, moderate levels of arousal characterized the LA-MOV condition, and low levels of arousal characterized the AT and CAL conditions. Taken together, these results, which were derived from subjective ratings, prove that the film stimuli that were used in this study successfully elicited the target positive emotions.

### Facial and Physiological Activities That Are Associated With the Emotional State of Being Moved

Corrugator EMG activity and the number of SCRs were higher in the HA-MOV condition than in the NE condition. The number of SCRs in the LA-MOV condition were higher compared to that in the NE condition and lower compared to that in the HA-MOV condition. Corrugator EMG activity in the LA-MOV condition was intermediate between HA-MOV and NE conditions, although statistical analyses did not directly support this result. These results indicate that the facial and physiological activities in the HA-MOV and LA-MOV conditions were characterized by similar response patterns. The emergent increase in corrugator EMG and electrodermal activities in response to the two moving films is consistent with the results of previous studies on chills that were evoked using music and the recitation of poems (Craig, 2005; Guhn et al., 2007; Grewe et al., 2009; Salimpoor et al., 2009, 2011; Mori and Iwanaga, 2014, 2017; Wassiliwizky et al., 2017). Therefore, when taken together with the results of analyses of subjective ratings, the present results indicate that the emotional responses that are associated with the emotional state of being moved in response to film clips is similar to those that are associated with music-induced chills in terms of facial muscle and electrodermal activities.

However, the results that were observed for HR diverged from past findings on music-induced chills. The change scores for HR in the HA-MOV and LA-MOV conditions did not differ from those in the NE condition. This result is inconsistent with past finding that the chills that are evoked by music are associated with an increase in HR (Grewe et al., 2009; Salimpoor et al., 2009, 2011). This discrepancy may be attributable to differences in the methods of emotion induction that were used in the past and present studies. Specifically, the findings of previous studies suggest that the induction of emotions using different types of stimuli (e.g., images, music, film clips) requires distinct attentional and cognitive processes that can affect facial and physiological activities (e.g., Cacioppo et al., 2000; Stemmler et al., 2001). Further, most of the previous studies have evoked chills using auditory stimuli such as music and the recitation of poems (Craig, 2005; Guhn et al., 2007; Grewe et al., 2009; Salimpoor et al., 2009, 2011; Mori and Iwanaga, 2014, 2017; Wassiliwizky et al., 2017). In contrast, in the present study, we used film clips to elicit emotions. The attentional and cognitive processes that are involved in stimulus processing can differ between music and film clips. In particular, film clips are known to demand greater attention because they entail dynamic displays that engage both the visual and auditory modalities (e.g., Rottenberg et al., 2007). It is well known that attention to the external environment causes cardiac deceleration as a result of the activation of the parasympathetic branch and sympathetic withdrawal (for a comprehensive review, see Bradley et al., 2012). In the present study, there was a decrease in HR from baseline to the time of film viewing in each film condition. This suggests that an attentional focus on the film stimuli caused a deceleration in HR. In other words, the high attentional demands of the film stimuli might have overpowered the effects of the emotional state of being moved and resulted in a deceleration in HR. Therefore, the anticipated increase in HR might not have emerged in the present study. This suggests that emotion induction methods may affect the emotional state of being moved at least when it is operationalized in terms of cardiac activity.

The present study also showed that the facial and physiological activities that were observed in the HA-MOV and LA-MOV conditions were characterized by similar response patterns. However, the intensities of these activities were higher in the HA-MOV condition than in the LA-MOV condition. The analytic results that were derived from arousal ratings showed that the HA-MOV film elicited higher levels of subjective arousal than the LA-MOV film. It has been established that SCRs reflect rapid fluctuations in eccrine sweat gland activity, which is caused by sympathetic nervous activation (Boucsein, 2012). Therefore, the present results that pertain to SCRs suggest that the HA-MOV film elicited higher levels of physiological arousal than the LA-MOV film. These results demonstrate that the film clips elicited different arousal levels for the emotional state of being moved and that the facial and physiological activities that are associated with being moved increase as a function of arousal level.

### Comparisons With Other Types of Positive Emotions

We also assessed typical facial and physiological responses to films that induce amusement, attachment, and calmness. The results revealed that zygomaticus EMG activity and number of SCRs were higher in the AMUSE condition than in the NE condition. On the other hand, corrugator EMG activity was lower in the AMUSE condition than in the NE condition. The increase in zygomaticus and electrodermal activities that result from the viewing of films that induce amusement has been repeatedly observed in previous studies (for a review, see Kreibig, 2010). Zygomaticus EMG activity was higher in the AT condition than that in the NE condition, whereas corrugator EMG activity was lower in the AT condition than in the NE condition. These results are consistent with past findings that the viewing of stimuli with baby schema (e.g., an infant’s face) increases zygomaticus EMG activity (e.g., Nittono and Ihara, 2017). The number of SCRs and change scores for HR did not differ between the AT and NE condition. This finding is also consistent with past findings that pictures that depict human or animal babies are associated with lower levels of arousal and fewer changes in physiological activities (Bradley et al., 2001; Sherman et al., 2009). Corrugator EMG activity was lower in the CAL condition than that in the NE condition, whereas SCRs and HR did not differ between the CAL and NE conditions. These results are also consistent with past findings that pictures and films that depict natural scenes are associated with lower levels of corrugator EMG activity (e.g., Bradley et al., 2001) and sympathetic withdrawal (for a review, see Kreibig, 2010).

The results of this study revealed that there are differences in the patterns of facial muscle activities that characterize the emotional state of being moved and other types of positive emotions: viewing the two moving films increased corrugator EMG activity, whereas viewing the other films decreased corrugator EMG activity. Given that valence ratings did not differ across the different positive-emotion conditions, the results that emerged for corrugator EMG activities clearly contradict past findings that corrugator muscle activity shares an inverse linear relationship with affective valence (e.g., Larsen et al., 2003). This discrepancy can be attributed to attentional and cognitive demands. It has been suggested that stimuli that elicit the emotional state of being moved is associated with intense involvement, cognitive interpretation, and focused attention (for a review, see Brattico et al., 2013). Previous studies have also reported that attentional and cognitive activities are associated with an increase in corrugator muscle activity (e.g., van Boxtel and Jessurun, 1993; Waterink and van Boxtel, 1994; Overbeek et al., 2014). Therefore, it is possible that the greater demand for attentional and cognitive resources that were necessitated by the viewing of moving films caused the observed increase in corrugator muscle activity. Another possible interpretation pertains to the emotional ingredients that constitute the feeling of being moved. Menninghaus et al. (2015) have suggested that the emotional state of being moved is associated with both positive and negative valence and that joy and sadness are the two key ingredients of the emotional state of being moved. It has been reported that sadness is associated with an increase in corrugator muscle activities (e.g., Schwartz et al., 1976). Therefore, it is possible that the increase in corrugator EMG activities that resulted from the viewing of the two moving films was caused by the induction of sadness. This interpretation is consistent with previous findings that the emotional state of being moved is associated with both positive and negative valence and that it can be categorized as a mixed emotional state (e.g., Menninghaus et al., 2015; for a review, see Zickfeld et al., 2019). Future studies should examine whether the facial muscle activities that are associated with the emotional state of being moved are distinct from those that are associated with pure sadness.

The change scores for HR did not differ across different film conditions. However, this does not necessarily lead to the conclusion that the cardiovascular activities that are associated with the emotional state of being moved are not distinct from those that are associated with other types of positive emotions. Previous studies have shown that other measures of cardiovascular activity such as cardiac output and total peripheral resistance demonstrate different response patterns for negative emotions (for a review, see Kreibig, 2010). Therefore, future studies should examine whether the cardiovascular activities that are associated with the emotional state of being moved are distinct from those that are associated with other types of positive emotions using different measures of cardiovascular activity such as blood pressure, cardiac output, and total peripheral resistance.

### Limitations and Future Directions

The present study demonstrated that film clips can elicit the emotional state of being moved and that it is characterized by an increase in corrugator muscle and electrodermal activities. However, it is important to note the several limitations of this study.

First, the size of the sample that was used in this study was relatively small, and it consisted of an unequal number of male and female participants. Consequently, this study lacked sufficient power to reliably examine potential moderators. In particular, sex may be an important moderator. It is well known that there are sex differences in the intensities and patterns of emotional responses (e.g., Kring and Gordon, 1998; Bradley et al., 2001; Overbeek et al., 2012). Therefore, future studies should use larger samples that consist of an equal number of male and female participants to examine sex differences in the film-elicited emotional state of being moved.

Second, the present results were derived from data that were obtained from young adults. However, previous studies have found that there are developmental changes in emotional responses (for a review, see Charles and Carstensen, 2010). Therefore, it is possible that other groups of individuals who are younger and older than the present sample will demonstrate different patterns of facial and physiological responses to moving films.

Third, we used only one film clip to induce each target emotion because long testing durations were expected to strain the participants. Film clips are widely used to elicit emotions in research studies (Rottenberg et al., 2007). However, film clips differ from each other on many potentially confounding characteristics (e.g., complexity, presence and number of human figures, presence or absence of background music, stimulus characteristics). It is noteworthy that, in the present study, the film clips that were used in the positive-emotion conditions had audio content, whereas the film clips that were used in the neutral condition did not have audio content; these differences may also explain the observed differences in facial and physiological activities across the different conditions. Therefore, the facial and physiological responses that were observed in this study may also be attributable to the confounding influence of stimulus characteristics rather than to emotions *per se*. In order to overcome this limitation, future research studies must use alternative research methodologies to test the generalizability of the present findings (e.g., by using several film clips to induce an emotion).

## Conclusion

The present study demonstrated that film clips can successfully induce the emotional state of being moved. In comparison to other types of positive emotions, the emotional state of being moved was characterized by an increase in corrugator muscle activity, which was modulated by the level of arousal of the emotional state of being moved. In contrast to past findings on music-induced chills, the emotional state of being moved was not associated with an increase in cardiac activity. This finding may be attributable to the greater attentional demands of film stimuli.

## Ethics Statement

This study was carried out in accordance with the recommendations of the Safety and Ethics Committee of the National Institute of Advanced Industrial Science and Technology with written informed consent from all subjects. All subjects gave written informed consent in accordance with the Declaration of Helsinki. The protocol was approved by the Safety and Ethics Committee of the National Institute of Advanced Industrial Science and Technology.

## Author Contributions

KK designed and performed the experiments, analyzed the data, and wrote the manuscript in consultation with SH, KS, and AO.

## Conflict of Interest Statement

The authors declare that the research was conducted in the absence of any commercial or financial relationships that could be construed as a potential conflict of interest.
